# Motor Imagery and Action Observation of Specific Neck Therapeutic Exercises Induced Hypoalgesia in Patients with Chronic Neck Pain: A Randomized Single-Blind Placebo Trial

**DOI:** 10.3390/jcm8071019

**Published:** 2019-07-12

**Authors:** Luis Suso-Martí, Jose Vicente León-Hernández, Roy La Touche, Alba Paris-Alemany, Ferran Cuenca-Martínez

**Affiliations:** 1 Motion in Brains Research Group, Institute of Neuroscience and Sciences of the Movement (INCIMOV), Centro Superior de Estudios Universitarios La Salle, Universidad Autónoma de Madrid, 28023 Madrid, Spain; 2 Department of Physiotherapy, Universidad CEU Cardenal Herrera, CEU Universities, 46115 Valencia, Spain; 3 Departamento de Fisioterapia, Centro Superior de Estudios Universitarios La Salle, Universidad Autónoma de Madrid, 28023 Madrid, Spain; 4 Instituto de Neurociencia y Dolor Craneofacial (INDCRAN), 28008 Madrid, Spain; 5 Instituto de Investigación Sanitaria del Hospital Universitario La Paz (IdiPAZ), 28046 Madrid, Spain

**Keywords:** motor imagery, action observation, chronic neck pain, pain modulation, pain neuroscience, musculoskeletal pain

## Abstract

The aim of the present study was to explore the pain modulation effects of motor imagery (MI) and action observation (AO) of specific neck therapeutic exercises both locally, in the cervical region, and remotely. A single-blind, placebo clinical trial was designed. A total of 30 patients with chronic neck pain (CNP) were randomly assigned to an AO group, MI group, or placebo observation (PO) group. Pain pressure thresholds (PPTs) of C2/C3, trapezius muscles, and epicondyle were the main outcome variables. Secondary outcomes included heart rate measurement. Statistically significant differences were observed in PPTs of the cervical region in the AO and MI groups between the preintervention and first postintervention assessment. Significant differences were found in the AO group in the epicondyle between the preintervention, first and second post-intervention assessments. Regarding heart rate response, differences were found in the AO and MI groups between the preintervention and average intervention measurements. AO and MI induce immediate pain modulation in the cervical region and AO also induces remote hypoalgesia. OA appears to lead to greater pain modulation as well as a greater heart rate response, however, both should be clinically considered in patients with CNP.

## 1. Introduction

Chronic neck pain (CNP) is a common musculoskeletal disorder with a high prevalence, and is the fourth leading condition that generates significant disability [1,2]. Patients with CNP usually present disturbances in postural control or neuromuscular control of the deep neck muscles associated with the onset of the condition [3,4]. Therefore, specific neck therapeutic exercise (SNTE) training of the deep neck musculature is widely used and might reduce pain and disability in patients with CNP compared with other types of conservative treatment [5].

SNTE has also been shown to induce immediate pain modulation, similar to the hypoalgesia induced by aerobic or isometric exercise [6,7]. Therefore, a central mechanism might be responsible for pain modulation after exercise [8]. On the other hand, the mental practice paradigms of motor simulation, such as action observation (AO) and motor imagery (MI), have recently been developed as a neurocognitive treatment tool for chronic pain [9,10]. MI is defined as a dynamic mental process of an action, without its actual motor execution [11]. AO evokes an internal, real-time motor simulation of the movements that the observer perceives visually [12]. Both mental practice paradigms trigger the activation of the neurocognitive mechanisms that underlie the planning and execution of voluntary movements in a manner that resembles how the action is performed in real life [13,14,15]. AO and MI might involve an autonomic nervous system (ANS) response. It has been shown that both MI and AO lead to changes in the ANS that cause sympathetic responses, and the neurophysiological base appears to be centrally controlled [16,17,18]. 

In recent years, both of these mental processes have been used in the acquisition of new motor gestures, range-of-motion enhancements, or for chronic pain management [19,20,21]. Despite the similarities of mental practice and exercise, it is uncertain whether MI or OA can induce immediate pain modulation in a similar manner as real exercise in patients with CNP, which would open new treatment approaches for these patients.

The aim of the present study was to explore the pain modulatory effects of MI and AO of SNTE in the cervical region. Our objective was to evaluate the hypoalgesic effects induced by MI and AO, both locally, in the cervical region, and remotely [6]. We hypothesized that MI and AO strategies would induce hypoalgesia and would be associated with an increase in heart rate, whereas a placebo observation (PO) did not.

## 2. Methods

### 2.1. Study Design

This study was a randomized, placebo clinical trial, with patient and evaluator blind, planned and conducted in accordance with Consolidated Standards of Reporting Trials (CONSORT) requirements, and was approved by a university ethics committee, with number CSEULS-PI-026/2019, Madrid, Spain.

This study was registered in the United States Randomized Trials Registry on clinicaltrial.gov (trial registry number: NCT03905577). All patients completed the informed consent document prior to the study.

### 2.2. Recruitment of Participants

The participants had been referred to the primary care physiotherapy service, had been diagnosed with CNP by their family doctor, and met the study’s inclusion criteria at one physiotherapy center. Participants were recruited between April 2019 and May 2019.

The inclusion criteria were as follows: (a) men and women aged between 18 and 65 years; and (b) a medical diagnosis of CNP with at least six months of neck pain symptoms. Exclusion criteria included the following: (a) patients with rheumatic diseases, cervical hernia or radicular pain, cervical whiplash syndrome, neck surgeries, or a history of arthrodesis; (b) systemic diseases; (c) vision, hearing, or vestibular problems; or (d) severe trauma or a traffic accident that had an impact on the cervical area.

All data were collected at the La Salle University Center for Advanced Studies. All the participants were given an explanation of the study procedures, which were planned according to the ethical standards of the Helsinki Declaration.

### 2.3. Randomization

Randomization was performed using a computer-generated random sequence table with a non-balanced three-block design (GraphPad Software, Inc., San Diego CA, USA). An independent researcher generated the randomization list, and a member of the research team who was not involved in the assessment of the participants or the intervention was in charge of the randomization and maintained the list. The patients included were randomly assigned to one of the three groups using the random sequence list, ensuring concealed allocation.

### 2.4. Blinding

The assessments and treatments were performed by various therapists. The evaluator was blinded to the participants’ group assignment. All the intervention procedures were performed by the same physiotherapist who had experience in the field and was blinded to the purpose of the study. Patients were blinded to their group allocation. In addition, a different researcher, blinded to the objectives of the study, performed the data analysis.

### 2.5. Interventions

#### 2.5.1. Action Observation Group

**Figure 1:**
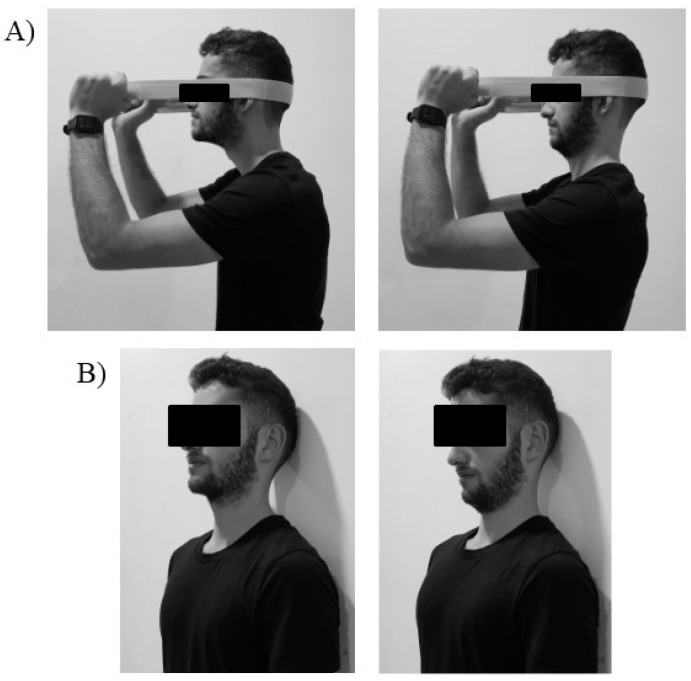
Specific therapeutic neck exercises included in the intervention. (**A**) Flexion-extension resistance exercise. (**B**) Cranio–cervical flexion exercise.

Patients in this group observed two SNTE typically used in the treatment of patients with CNP. Both exercises were based on the motor gesture of craniocervical flexion (Figure 1). Patients in the AO group performed the observation through a video of the continuous performance of both exercises repeatedly during two series of 1 minute for each exercise, with a total duration of 4 minutes. The participants were seated with a laptop in front of them.

The first exercise involved a resistance deep muscle contraction by performing continuous the cranio-cervical flexo-extension gesture with the resistance of an elastic band (Figure 1A). The second exercise consisted of maintaining the cervical spine in a neutral position in a sitting position and performing a deep muscle contraction to flatten the curve of the neck by nodding with the head. This task involves flexion of the cranium on the cervical spine with the deep cervical muscle contraction (Figure 1B). Patients were instructed to just observe both movements on the monitor without executing or imagining any movement.

#### 2.5.2. Motor Imagery Group

The patients in this group performed a motor imagery protocol of the same cervical exercises observed by the AO group (Figure 1). Patients were instructed on the movements they had to imagine by showing both exercises and the auditive precise instructions for each movement during the intervention. Next, they were instructed to perform a guided third-person mental task of visual motor imagery. For this intervention, the participants were guided by the therapist to imagine the SNTE, trying to form a visual mental image or picture of both movements and attempting to visualize the movement as clear and vivid as possible. The MI intervention of both exercises was performed during two series of 1 minute for each exercise, with a total duration of 4 minutes.

#### 2.5.3. Placebo Observation Group

Patients in this group underwent a PO protocol. A video composed of only nature landscape clips was visualized for 4 minutes, without visualizing any motor gesture. This kind of PO protocol has been used in previous research [22,23].

### 2.6. Outcomes

#### 2.6.1. Primary Outcomes

##### Pressure Pain Thresholds

A pressure pain threshold (PPT) is defined as the minimal amount of pressure at which a sense of pressure first changes to pain. The mechanical pressure algometer (Wagner Instruments, Greenwich, CT, USA) used in this study consisted of a round rubber disk (area 1 cm^2^) attached to a pressure (force) gauge. The gauge displayed values in kilograms, but because the surface of the rubber tip was 1 cm^2^, the readings were expressed in kg/cm^2^. The range of the pressure algometer values was from 0 to 10 kg, in 0.1 kg intervals. The pressure was applied at a rate of 0.31 kg/second [24]. Previous studies have reported an intraexaminer reliability of this procedure ranging from 0.6–0.97, whereas the interexaminer reliability ranged from 0.4–0.98 [25]. 

PPTs were tested in four different locations. These sites included the angle of both the upper fibers of the left and right trapezius muscles (5–8 cm superior medial from the superior angle of the scapula), the zygapophyseal joint of C2/C3, and the nondominant lateral epicondyle. All the assessments were performed in a quiet room. In order to familiarize the participants with the test procedure, pressure was first applied to an area that would not be tested during the study. Three consecutive measurements of the PPT at the four locations at intervals of 30 seconds and the mean of these three trials was used for the data analysis [25].

#### 2.6.2. Secondary Outcomes

##### Heart Rate

Heart rate (HR) was measured to determine how the patients were engaging in the interventions, because HR is under autonomic nervous system control. The heart rate was recorded to quantify the changes produced during the performance of the mental motor practice. The Garmin Forerunner VR 225 is a commercially available wrist-worn heart rate monitor that uses an optical green light sensor to detect pulse rate, which represents HR. The Garmin Forerunner VR 225 was programmed with the participants’ sex, age, weight, and height, and was fitted on the left forearm, according to the user manual. Previous studies have shown moderate to strong validity of the Garmin Forerunner VR 225 versus traditional electrocardiography measures (Pearson *r* = 0.650–0.868).

##### Motor Imagery Ability 

The movement imagery questionnaire—revised (MIQ-R) is an eight-item self-report inventory used to assess visual and kinesthetic motor imagery ability. Four different movements are included in the MIQ-R, which is comprised of four visual and four kinesthetic items. For each item, participants read a description of the movement. They then physically performed the movement and were instructed to resume the starting position after finishing the movement and before performing the mental task, which was to imagine the movement visually or kinesthetically. Next, each participant rated the ease or difficulty of generating the mental image on a seven-point scale, in which 7 indicated “very easy to see/feel” and 1 “very difficult to see/feel.” The internal consistencies of the MIQ-R have been adequate, with Cronbach’s α coefficients ranging above 0.84 for the total scale, 0.80 for the visual subscale, and 0.84 for the kinesthetic subscale [26].

##### Mental Chronometry 

Mental chronometry (MC) is a reliable measure that has been widely used to record objective measurements of the ability to create mental motor images [27,28,29]. To assess MC, the time dedicated to imagining each task of MIQ-R questionnaire was first recorded using a stopwatch. The time interval between the command to start the task (given by the evaluator) and the verbal response at the conclusion of the task (given by the participant) was recorded. After the motor imagery task, the participants were asked to execute the real movement of the task, and the time dedicated to performing each task was recorded using a stopwatch. Both time measurements were taken to obtain the temporal congruence between both tasks. During motor imagery, spatial and temporal information were similar to those of the physical execution, suggesting that the time taken to imagine the movement would be similar to that needed for its real execution. MC was used to measure the temporal congruence between real and imagined movements [28,30]. 

##### Pain-related Fear of Movement

Pain-related fear of movement was assessed using the 11-item Spanish version of the Tampa Scale for Kinesiophobia, whose reliability and validity have been demonstrated [31]. The Tampa Scale for Kinesiophobia consists of two subscales, one related to fear of activity and the other related to fear of harm. The final score can range between 11 and 44 points, with higher scores indicating greater perceived kinesiophobia [31].

##### Pain Catastrophizing

The Spanish version of the Pain Catastrophizing Scale assesses the degree of pain catastrophizing and is a reliable and valid form of measurement. It is composed of 13 items, with a three-factor structure of rumination, magnification, and helplessness that must be answered with a numeric value between 0 (not at all) and 4 (all the time), with a maximum score of 52 points, with higher scores indicating greater pain catastrophizing [32].

##### Neck Disability

Disability was measured using the Spanish-validated Neck Disability Index (NDI), which consists of 10 items related to daily functional activities. Each question is measured on a scale from 0 (no disability) to 5, and an overall score out of 100 is calculated by adding each item score together and multiplying it by two. A higher NDI score indicates a patient’s greater perceived disability due to neck pain. It has been shown to have high “test–retest” reliability and to have appropriate psychometric properties [33].

##### Physical Activity Level

The level of physical activity was objectified through the International Physical Activity Questionnaire, which allows the participants to be divided into three groups according to their level of activity: high, moderate, and low or inactive [34]. This questionnaire has shown acceptable validity and psychometric properties for measuring total physical activity.

##### Visual Analogue Scale

A visual analogue scale (VAS) was used to measure pain intensity. The VAS is a 100-mm line with two endpoints representing the extreme states “no pain” and “the maximum pain imaginable”. It has been shown to have good retest reliability (r = 0.94, *p* < 0.001) [35,36].

### 2.7. Procedures

Each participant completed an informed consent document to participate in the study, in addition to a set of questionnaires to complete before starting the intervention. These questionnaires included psychometrics forms and a questionnaire about age, sex, medication, anthropometric measures, pain duration, and the predominant pain location. The psychological variables were evaluated with self-assessments and the pain intensity by VAS. Then, MIQ-R and mental chronometry were assessed. The preintervention PPT measurements were made at the four sites by an external assessor, in random order. Subsequently, an initial HR measurement was performed. The Garmin Forerunner VR 225 monitor was placed, the patients lay down for five minutes, and then sat upright for two more minutes. In both positions, the patients were instructed to maintain a comfortable position and relaxed breathing, with the aim of obtaining a baseline HR measurement. The first measurement was taken at the end of seven minutes, just before the start of the intervention (preintervention measure). At this time and in a sitting position, patients performed the AO protocol, MI or PO, according to the randomized group. HR measurements were taken during the intervention. A measurement was recorded every 15 seconds for four minutes; subsequently, the average of all the measurements was recorded (intervention average measure). The postintervention HR was recorded at the end of the four minutes of the intervention (postintervention measurement). Immediately after the intervention, a blinded evaluator measured the PPTs in all four locations (post-1). Following this, patients were asked to sit relaxed and comfortably, without movement, for 10 minutes, and the PPTs were again measured (post-2).

### 2.8. Statistical Analysis

The statistical data analysis was performed using statistical SPSS software version 22.0 (SPSS Inc., Chicago, IL, USA). The normality of the variables was evaluated by the Shapiro–Wilk test. Descriptive statistics were used to summarize the data for continuous variables and are presented as mean ± standard deviation, 95% confidence interval. Additionally, we compared age, weight, and height between groups with a one-way ANOVA to explore whether the groups were homogeneous at baseline. The chi-squared test was used for the categorical variables that were presented as frequency and percentage. A mixed model analysis of variance (ANOVA) was conducted to study the effect of the between-participant “treatment group” factor in each of the three categories (AO, MI, and placebo) and the within-participant “time” factor, also in each of the three categories (i.e., pre-, post-1, and post-2), of all the dependent variables except for the HR. For the HR, the difference between the preintervention measurement, average intervention measurement, and the immediate postintervention measurement was evaluated (pre-, average intervention, post-1). A post hoc analysis with Bonferroni correction was performed in the case of significant ANOVA findings for multiple comparisons between variables. Effect sizes (*d*) were calculated according to Cohen’s method, in which the magnitude of the effect was classified as small (0.20–0.49), moderate (0.50–0.79), or large (>0.8) [37]. The α level was set at 0.05 for all tests. 

## 3. Results

**Figure 2:**
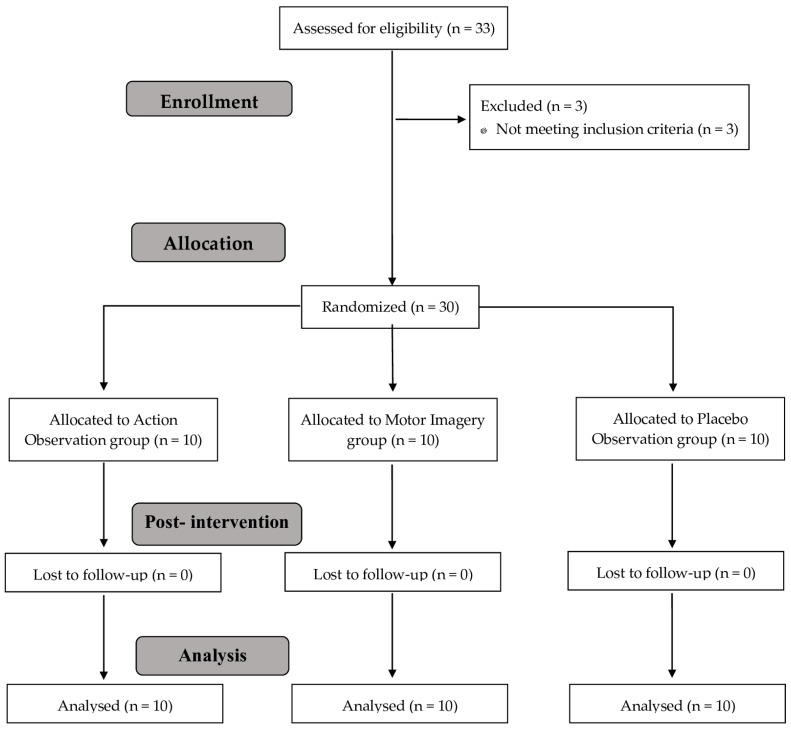
Study flow chart.

A total of 30 patients with CNP were included and were randomly allocated into three groups of 10 participants per group (Figure 2). There were no adverse events reported in either group. All the variables presented a normal distribution. No statistically significant differences were found between groups for any of the primary variables, demographic data, or self-report variables at baseline between the groups, except for educational level (*p* < 0.05) (Table 1 and Table 2).

### 3.1. Primary Outcomes

#### 3.1.1. Pressure Pain Threshold

##### C2/C3 

**Figure 3:**
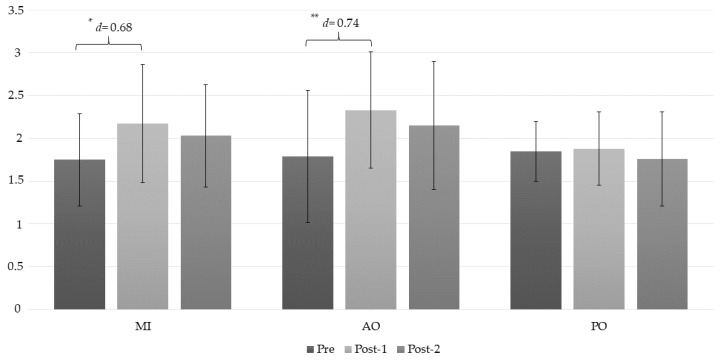
Changes in the pressure pain threshold (PPT) C2/C3 measurement. *: *p* < 0.05; **: *p* < 0.001; AO: action observation; MI: motor imagery group; PO: placebo observation group; Pre: pre-intervention measurement; Post-1: first post intervention measurement (immediately after intervention); Post-2: second post intervention measurement (10 min after intervention).

The ANOVA revealed significant changes in the C2/C3 PPT measurement during group*time (*F* = 3.04, *p* = 0.025, ƞ^2^ = 0.185) and time (*F* = 10.74, *p* < 0.01, ƞ^2^ = 0.285). The post hoc analysis revealed significant intragroup differences (Table 3). Statistically significant differences were observed between the preintervention assessment and the post-1 intervention in the AO and MI groups, with a moderate effect size (*p* < 0.001, *d* = 0.74, and *p* = 0.004, *d* = 0.68, respectively) (Figure 3).

##### Right Trapezius Muscle

**Figure 4:**
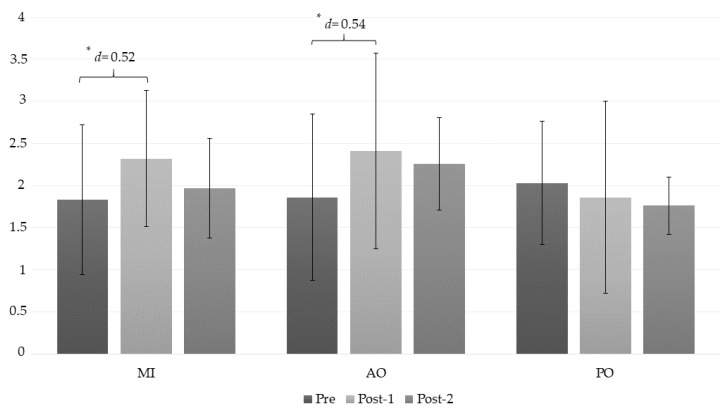
Changes in the PPT right trapezius measurement. *: *p* < 0.05; AO: action observation; MI: motor imagery group; PO: placebo observation group; Pre: pre-intervention measurement; Post-1: first post intervention measurement (immediately after intervention); Post-2: second post intervention measurement (10 min after intervention).

The ANOVA revealed significant changes in the right trapezius muscle PPT measurement during group*time (*F* = 3.42, *p* = 0.014, ƞ^2^ = 0.202) and time (*F* = 4.75, *p* = 0.013, ƞ^2^ = 0.15) The post hoc analysis revealed significant intragroup differences (Table 3). Statistically significant differences were observed between the preintervention assessment and the post-1 intervention in the AO and MI groups, with a moderate effect size (*p* = 0.012, *d* = 0.54, and *p* = 0.028, *d* = 0.52, respectively) (Figure 4).

##### Left Trapezius Muscle

**Figure 5:**
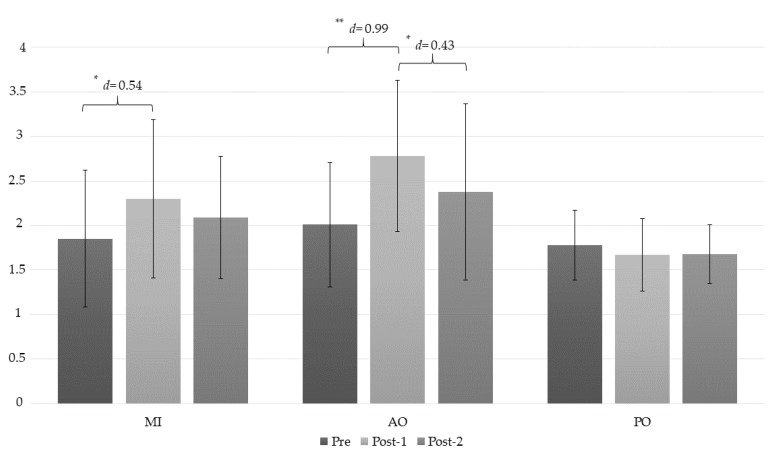
Changes in the PPT left trapezius measurement. *: *p* < 0.05; **: *p* < 0.001; AO: action observation; MI: motor imagery group; PO: placebo observation group; Pre: pre-intervention measurement; Post-1: first post intervention measurement (immediately after intervention); Post-2: second post intervention measurement (10 min after intervention).

The ANOVA revealed significant changes in the left trapezius muscle PPT measurement during group*time (*F* = 4.16, *p* = 0.005, ƞ^2^ = 0.235) and time (*F* = 8.92, *p* < 0.001, ƞ^2^ = 0.248). The post hoc analysis revealed significant intragroup differences between the preintervention assessment and the post-1 measurement, with a large effect size (*p* < 0.001, *d* = 0.99), and between the post-1 and the post-2 assessments in the AO group, with a moderate effect size (*p* = 0.037, *d* = 0.43) (Table 3). In addition, statistically significant differences were observed in the MI group between the preintervention assessment and the post-1 measurement, with a moderate effect size (*p* = 0.015, *d* = 0.54). Finally, statistically significant differences were found between the AO and PO groups, with a large effect size (*p* < 0.001, *d* = 1.66) (Figure 5).

##### Lateral Epicondyle

**Figure 6:**
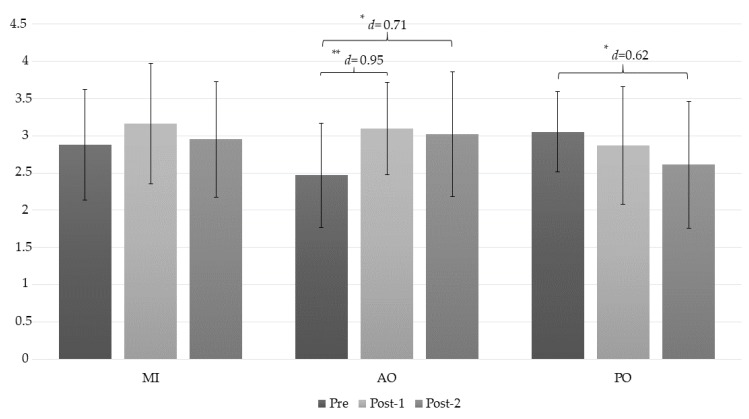
Changes in the PPT epicondyle measurement. *: *p* < 0.05; **: *p* < 0.001; AO: action observation; MI: motor imagery group; PO: placebo observation group; Pre: pre-intervention measurement; Post-1: first post intervention measurement (immediately after intervention); Post-2: second post intervention measurement (10 min after intervention).

The ANOVA revealed significant changes in the lateral epicondyle PPT measurement during group*time (*F* = 6.4, *p* < 0.001, ƞ^2^ = 0.321) and time (*F* = 4.44, *p* = 0.016, ƞ^2^ = 0.141). The post hoc analysis revealed significant intragroup differences only in the AO group (Table 3). Statistically significant differences were observed between the preintervention assessment and the post-1 measurement, with a large effect size (*p* < 0.001, *d* = 0.95), and between the pre-intervention assessment and the post-2 measurement, with a moderate effect size (*p* = 0.005, *d* = 0.71). In addition, intra-group differences were found in the PO group between the preintervention and post-2 intervention measurements, with a moderate effect size (*p* = 0.032, *d* = 0.62) (Figure 6).

### 3.2. Secondary Outcomes

#### Heart Rate

The ANOVA revealed significant changes in heart rate during group*time (*F* = 18.52, *p* < 0.001, ƞ^2^ = 0.578) and time (*F* = 85.74, *p* < 0.001, ƞ^2^ = 0.761). The post hoc analysis revealed significant intragroup differences in the MI and AO groups between the preintervention assessment and the intervention average assessment (*p* < 0.001 in both groups, *d* = 0.48 and *d* = 0.67, respectively). Statistically significant differences were observed between the preintervention assessment and the postintervention measurement, with a large effect size in the AO and MI groups (*p* < 0.001 in both groups, *d* = 1.3 and *d* = 0.84, respectively). In addition, in both groups, statistically significant differences were found between the intervention average measurement and postintervention measurement (*p* < 0.001 in both groups, *d* = 0.42 and *d* = 0.7, respectively).

Statistically significant intergroup differences were found between the AO and PO groups in the intervention average measurement (*p* < 0.001; *d* = 1.4). In addition, significant intergroup differences were found in the postintervention measurement between the MI and AO groups, with a large effect size (*p* = 0.042, *d* = 1.10), and between the AO and PO groups (*p* = 0.001, *d* = 1.92) (Table 4).

## 4. Discussion

The aim of the present study was to explore the immediate modulatory pain effects of MI and AO of SNTE in the cervical and remote regions. Our results show that both MI and AO induced an immediate pain modulation response in the cervical region (post-1), however, it was not sustained in the second measurement after the intervention. In the epicondyle, only AO induced pain reduction between the preintervention measurement and both postintervention measurements. AO and MI interventions provoked an increase in HR, however, AO showed significant differences in comparison with the PO and MI groups.

Exercise-induced hypoalgesia is a well-documented phenomenon. Although most research has demonstrated modulating effects on pain by aerobic exercise, O’Leary et al. have shown that performing SNTEs, similar to those employed in the present study, produced local hypoalgesic responses in the cervical region [6]. According to the literature, AO and MI might provoke cortical activations similar to the real movement execution; thus, it is possible that the overlapping of cortical areas between real execution and mental practice could explain our findings [38,39]. In this regard, Beinert et al. found no differences in PPTs between performing and imagining motor control exercises of the flexor neck musculature in patients with neck pain. These data suggest that there is probably a top-down central mechanism responsible for hypoalgesia, according to our results [40]. However, Beinert et al. found no differences in the PPTs of the cervical region after an MI or AO intervention of the articular position error task [41]. These controversial data appear to be related to the imagined or observed task. It is possible that if the selected movement is able to trigger pain or fear responses in patients during real execution, the pain modulation response might be lower if it is performed mentally. This result has also been found in studies using functional magnetic resonance, that show the activation of cortical areas related to pain processing after the mental practice of painful movements [42]. In this regard, Forkmann et al. examined the relationship between painful stimuli and cortical encoding of visual stimuli [43]. Their results showed that when a visual stimulus was accompanied with a painful input, there was a decrease in the activity of the hippocampus associated with a lower encoding of the visual stimulus. It is possible that if an imagined or observed painful movement activates brain areas similar to a real painful stimulus, the coding of visual information might also be influenced, affecting pain modulation. 

In addition to the pain-trigger responses, another relevant factor could be pain-related fear. Previous research has shown that high levels of fear of movement directly affect the periaqueductal gray through the amygdala, which might have a direct negative effect on endogenous pain modulation [44]. The study by de-la-Puente-Ranea et al. showed hypoalgesic responses after complete cervical rotation movements in patients with CNP, although this movement could be considered painful or fear-associated in these patients [45]. However, the levels of patients’ fear of movement were low, and it is possible that low fear of movement levels could influence these results in a manner opposite to the aforementioned findings of Beinert et al. We therefore suggest that MI and AO might produce relevant hypoalgesic responses, but it is necessary to consider factors such as pain-related fear or the possible pain-trigger responses related to the imagined or observed movement.

A relevant finding of the present study is that MI and AO produced pain modulation responses compared to PO. This finding is important because previous studies have suggested that distraction might be a mechanism involved in pain modulation produced by mental practice [46,47]. Although in the present study no immersive strategies were used that could provoke greater distraction, other mechanisms are required to explain the hypoalgesia induced by mental practice. In addition to the aforementioned top-down mechanism, additional hypotheses have been proposed concerning interactions between pain modulation and heart rate, which were also found in this study, suggesting a systemic pain modulatory effect. Previous research has investigated manual therapy hypoalgesia models, showing that hypoalgesia is related to increased ANS activity [48,49]. In addition, patients with chronic pain experience maladaptive neuroplastic changes that could lead to impaired cortical-motor representation and diminished cortical excitability [50,51]. In this regard, previous studies have shown that both AO and MI can cause an increase in cortical representation and excitability, influencing areas such as the primary motor cortex or the dorsal premotor cortex [52,53]. Larsen et al. showed that MI and AO could induce an increase in cortical excitability, which was associated with a decrease in pain perception [54]. These findings are consistent with those obtained by Volz et al., in which pain modulation was observed after AO training, which was associated with increased cortical excitability of the motor cortex. This outcome is also directly related to the neural networks related to pain modulation through corticothalamic networks, as well as changes in neural plasticity [55,56].

On the other hand, our results showed that the AO provoked greater local and remote hypoalgesic responses and triggered a higher HR increase compared with MI. Possible differences between AO and MI remain unclear and more research is needed. HR is under autonomic control, which could give an estimate of the physiological responses produced by both interventions, although other measurements, such as skin conductance or temperature, are necessary to establish whether AO or MI caused increased activity of the autonomic nervous system. However, one of the main difficulties in interventions with mental motor practice is to know if the patient was engaging to the intervention in the correct form. Our HR date showed that in both groups, patients were engaged in the intervention, although in the AO group the HR increase was higher compared to the MI group. One potential factor that could influence this outcome is the exercises selected for the intervention. The selection of these exercises was based on their extensive clinical application, the pain modulation effects found with their real execution, and the intent to prevent fear in their execution. Fear responses to movements perceived as dangerous have been associated with increases in ANS activity and pain intensity [57,58]. However, a significant point to note is that MI requires a good ability to imagine and is less effective in people with poorer imaginative ability [59]. Some aspects, such as imagining the body segment movement, the complexity or familiarity of the movement, as well as levels of physical activity, have all been related to MI performance ability. SNTE exercises are highly difficult to imagine, due to the fact that they require motor learning of unknown, complex, and high precision movements. This could result in less mental effort performed by patients in the MI group, due to their inability to imagine the exercises, and less effort is associated with decreased ANS responses and might therefore be associated with decreased hypoalgesic responses [60,61]. Another hypothesis in this aspect is that the difficulty in imagining the exercises could provoke a mental stress in the patients of the MI group that could be related to the hypoalgesia. The stress-inducing hypoalgesia phenomenon has been previously reported in the scientific literature and may be an alternative explanation to the results obtained [62]. In addition, patients with chronic pain have a decreased ability to create mental motor images, which could also be related to our results [63]. 

### 4.1. Clinical Implications

The results of the present study showed that AO and MI could provoke pain modulatory effects in the cervical region. The implementation of mental practice in patients with persistent pain, is highly relevant, as they could be performed in clinical environments where in the early stages, it is not possible to perform motor gestures in a real way due to the presence of pain or psychosocial variables, like fear of movement. These tools offer opportunities to improve the different stages of rehabilitation for patients with dysfunctional and maladaptive pain. In addition, this approach could increase the effectiveness of the current treatments, thus, they should be considered due to their simple implementation and cost-effectiveness in everyday daily routines or clinical practice. In addition, mental practice may have additional positive effects on motor learning or increase patient adherence to exercising the rehabilitation process. Future studies should continue to investigate the benefits of AO and MI in patients with chronic pain, as well as their implementation in clinical practice.

### 4.2. Limitations

This study presents some limitations. First, the sample size was small, and thus, the results should be considered with caution. In addition, the results have only been considered in the short term, and the duration and type of intervention might have been insufficient for greater increases in pain modulation in patients with CNP, especially in the MI group. Second, changes in clinical pain were not evaluated. Longer mental practice interventions may determine changes in clinical pain, which is certainly a very relevant aspect. More research is needed to determine the role of mental practice in pain modulation in patients with chronic pain.

## 5. Conclusions

Both the AO and MI of specific neck exercises are able to induce immediate pain modulation of the cervical region. Although both strategies led to increases in PPTs, AO appears to have led to greater local and remote pain modulation, as well as a greater response from the ANS. More research is needed in this area on the role and additional benefits of mental practice in terms of pain modulation and its implementation in clinical practice.

## Figures and Tables

**Table 1:** Descriptive statistics of sociodemographic data.

Measures	AO Group (*n* = 10)	MI Group (*n* = 10)	PO Group (*n* = 10)	*p* Value
**Age**	33.5 ± 14.25	30.6 ± 11.53	27.70 ± 6.39	0.520
**Height** (cm)	171.9 ± 0.80	173.10 ± 0.70	174 ± 0.40	0.798
**Weight** (kg)	66.7 ± 7.97	68.70 ± 4.8	69.5 ± 8.26	0.672
**Pain intensity** (VAS)	68.9 ± 13.95	75 ± 7.73	70.8 ± 9.36	0.437
**Pain duration** (month)	27.9 ± 17.99	26.2 ± 12.45	17.4 ± 10.05	0.212
**Sex**				0.875
Male	5 (50)	5 (50)	4 (40)	
Female	5 (50)	5 (50)	6 (60)	
**Educational Level**				0.03
Secondary education	3 (30)	5 (50)	0 (00)	
College education	7 (70)	5 (50)	10 (100)	
**Marital Status**				0.136
Single	7 (70)	3 (30)	5 (50)	
Married	3 (30)	4 (40)	4 (40)	
Divorced	0 (0)	3 (30)	1 (0)	
**Pain Location**				0.530
Right	5 (50)	2 (20)	2 (20)	
Left	3 (30)	5 (50)	4 (40)	
Both	2 (20)	3 (30)	4 (40)	

Values are presented as mean ± standard deviation or number (%); MI: motor imagery; AO: action observation; PO: placebo observation; VAS: visual analogue scale.

**Table 2:** Descriptive statistics of self-reported and psychosocial data.

Measures	AO Group (*n* = 10)	MI Group (*n* = 10)	PO Group (*n* = 10)	*p* Value
**PCS**	31 ± 5.9	32.2 ± 6.71	33.1 ± 5.65	0.745
**TSK-11**	32.3 ± 6	33 ± 4.85	31.3 ± 3.93	0.633
**NDI**	30.5 ± 3.62	29.8 ± 3.82	32.1 ± 4.48	0.430
**IPAQ**	1760.6 ± 483.51	1713.85 ± 500.3	1785.7 ± 659.17	0.958
**MIQ-R**	47.4 ± 4.77	47.3 ± 7.86	48 ± 4.52	0.960
**MC**	3.65 ± 3.96	4.39 ± 5.7	4.71 ± 4.52	0.879

Values are presented as mean ± standard deviation or number (%); MI: motor imagery; AO: action observation; PO: placebo observation; PCS: Pain Catastrophizing Scale; TSK: Tampa Scale of Kinesiophobia; NDI: Neck Disability Index; IPAQ: International Physical Activity Questionnaires; MIQ-R: Movement Imagery Questionnaire-Revised; MC: Mental Chronometry.

**Table 3:** Results of the PPT outcomes.

Measure	Group	Mean ± SD			Mean Difference (95% CI); Effect Size (*d*) (a) Pre–Post 1 (b) Pre–Post 2 (c) Post 1–Post 2
		Pre	Post-1	Post-2	
**PPT** **(C2/C3)**	**MI**	1.75 ± 0.54	2.17 ± 0.69	2.03 ± 0.60	(a) −0.41 * (−0.71 to 0.12); *d* = 0.68 (b) −0.27 (−0.66 to 0.11); *d* = 0.49 (c) 0.14 (−0.83 to 0.37); *d* = 0.21
	**AO**	1.79 ± 0.77	2.33 ± 0.68	2.15 ± 0.75	(a) −0.54 ** (−0.84 to −0.25); *d* = 0.74 (b) −0.36 (−0.75 to 0.02); *d* = 0.47 (c) 0.18 (−0.05 to 0.40); *d* = 0.25
	**PO**	1.85 ± 0.35	1.88 ± 0.43	1.76 ± 0.55	(a) −0.02 (−0.02 to 0.75); *d* = 0.07 (b) 0.09 (0.29 to 0.48); *d* = 0.19 (c) 0.11 (−0.11 to 0.33); *d* = 0.24
**Mean difference (95% CI)** **Effect size (*d*)**					
**MI-AO**		−0.03 (−0.69 to 0.63); *d* = 0.06	−0.16 (−0.86 to 0.54); *d* = 0.23	−0.13 (− 0.85 to 0.60); *d* = 0.17	
**MI-PO**		−0.10 (−0.76 to 0.56); *d* = 0.22	0.29 (−0.41 to 0.99); *d* = 0.5	0.27 (−0.46 to 0.99); *d* = 0.47	
**AO-PO**		−0.07 (−0.73 to 0.59); *d* = 0.1	0.46 (−0.24 to 1.11); *d* = 0.79	−0.39 (−0.34 to 1.12); *d* = 0.59	
**PPT** **(RT)**	**MI**	1.83 ± 0.89	2.32 ± 0.99	1.97 ± 0.73	(a) −0.49 * (−0.93 to −0.04); *d* = 0.52 (b) −0.14 (−0.60 to 0.32); *d* = 0.17 (c) 0.35 (−0.22 to 0.72); *d* = 0.40
	**AO**	1.86 ± 0.81	2.41 ± 1.16	2.26 ± 0.1.14	(a) −0.55 * (−0.99 to −0.11); *d* = 0.54 (b) −0.40 (−0.86 to 0.07); *d* = 0.40 (c) 0.16 (−0.21 to 0.52); *d* = 0.13
	**PO**	2.03 ± 0.59	1.86 ± 0.55	1.76 ± 0.34	(a) 0.17 (−0.28 to 0.61); *d* = 0.29 (b) 0.27 (−0.19 to 0.74); *d* = 0.56 (c) 0.11 (−0.26 to 0.47); *d* = 0.21
**Mean difference (95% CI)** **Effect size (*d*)**					
**MI-AO**		−0.02 (−0.91 to 0.86); *d* = 0.03	−0.09 (−1.15 to 0.98); *d* = 0.08	0.28 (−1.2 to 0.63); *d* = −0.40	
**MI-PO**		−0.20 (−1.08 to 0.68); *d* = 0.26	0.46 (−0.61 to 1.52); *d* = 0.57	0.21 (−0.70 to 1.13); *d* = 0.36	
**AO-PO**		−0.18 (−1.06 to 0.71); *d* = 0.23	0.54 (−0.52 to 1.61); *d* = 0.6	0.49 (−0.42 to 1.41); *d* = 0.59	
**PPT** **(LT)**	**MI**	1.85 ± 0.77	2.30 ± 0.89	2.09 ± 0.69	(a) −0.46 * (−0.85 to −0.07); *d* = 0.54 (b) −0.24 (−0.66 to 0.17); *d* = 0.32 (c) 0.21 (−0.17 to 0.60); *d* = 0.26
	**AO**	2.01 ± 0.70	2.78 ± 0.85	2.38 ± 0.99	(a) −0.78 ** (−1.16 to −0.39); *d* = 0.99 (b) −0.37 (−0.79 to 0.04); *d* = 0.43 (c) −0.40 * (0.02 to 0.79); *d* = 0.43
	**PO**	1.78 ± 0.39	1.67 ± 0.41	1.68 ± 0.33	(a) 0.10 (−0.28 to 0.49); *d* = 0.27 (b) 0.09 (−0.32 to 0.51); *d* = 0.27 (c) 0.01 (−0.39 to 0.38); *d* = 0.02
**Mean difference (95% CI)** **Effect size (*d*)**					
**MI-AO**		−0.16 (−0.90 to 0.57); *d* = 0.21	−0.48 (−1.34 to 0.38); *d* = 0.55	−0.29 (−1.12 to 0.53) *d* =0.33	
**MI-PO**		−0.06 (−0.67 to 0.80) *d* = 0.11	0.63 (−0.23 to 1.49), *d* = 0.90	0.40 (−0.42 to 1.22); *d* = 0.75	
**AO-PO**		0.23 (0.51 to 0.96); *d* = 0.40	1.11 ** (0.25 to 1.96); *d* = 1.66	0.69 (−0.13 to 1.51); *d* = 0.94	
**PPT** **(Epicondyle)**	**MI**	2.88 ± 0.74	3.16 ± 0.81	2.95 ± 0.78	(a) −0.29 (−0.60 to −0.01); *d* = 0.36 (b) −0.08 (−0.21 to 0.62); *d* = 0.09 (c) 0.21 (−0.21 to 0.62); *d* = 0.26
	**AO**	2.47 ± 0.70	3.1 ± 0.62	3.02 ± 0.84	(a) −0.64 ** (−0.95 to −0.33); *d* = 0.95 (b) −0.56 * (−0.96 to −0.15); *d* = 0.71 (c) 0.07 (−0.49 to 0.34); *d* = 0.11
	**PO**	3.05 ± 0.54	2.87 ± 0.79	2.61 ± 0.85	(a) 0.18 (−0.13 to 0.49); *d* = 0.26 (b) 0.44 * (0.03 to 0.84); *d* = 0.62 (c) 0.25 (−0.67 to 0.16); *d* = 0.31
**Mean difference (95% CI)** **Effect size (*d*)**					
**MI-AO**		0.41 (−0.35 to 1.16); *d* = 0.56	−0.06 (−0.79 to 0.91); *d* = 0.08	−0.07 (−1.01 to 0.87) *d* = 0.08	
**MI-PO**		−0.18 (−0.94 to 0.58) *d* = 0.26	0.30 (−0.55 to 1.15), *d*= 0.36	0.34 (−0.59 to 1.28); *d* = 0.41	
**AO-PO**		−0.58 (−1.34 to 0.18); *d* = 0.92	0.24 (−0.61 to 1.09); *d* = 0.32	0.41 (−0.53 to 1.35); *d* = 0.48	

* *p* < 0.05; ** *p* < 0.001. AO: action observation group; MI: motor imagery group; PO: placebo observation group; PPT: pressure pain threshold; RT: right trapezius measurement; LT: left trapezius measurement; pre: preintervention measurement; Post-1: first post intervention measurement (immediately after intervention); Post-2: second post intervention measurement (10 min after intervention).

**Table 4:** Results of heart rate measurement.

Measure	Group	Mean ± SD			Mean Difference (95% CI); Effect Size (*d*). (a) Pre-Intervention (b) Pre–Post (c) Intervention–Post
		Pre	Intervention	Post	
**HR**	**MI**	72.3 ± 5.38	74.84 ± 4.99	77.3 ± 6.4	(a) −2.54 ** (−4.09 to −0.97) *d* = 0.48 (b) −5 ** (−7.15 to −2.85); *d* = 0.84 (c) −2.47 ** (−3.74 to −1.2) *d* = 0.42
	**AO**	75.7 ± 6.77	80.08 ± 6.24	84.8 ± 7.19	(a) −4.38 ** (−5.94 to −2.82) *d* = 0.67 (b) −9.1 ** (−11.24 to −6.95); *d* = 1.3 (c) −4.72 ** (−5.99 to −3.45) *d* = −0.7
	**PO**	71.6 ± 5.42	72.12 ± 5.05	72.6 ± 5.4	(a) −0.52 (−2.08 to 1.04) *d* = −0.09 (b) −1 (−0.73 to 2.73); *d* = −0.18 (c) −0.48 (−1.75 to 0.79); *d* = −0.09
**Mean difference (95% CI)** **Effect size (*d*)**					
**MI-AO**		−3.4 (−10.13 to 3.33); *d* = −0.55	−5.24 (−11.48 to 0.99); *d* = −0.92	−7.5 * (−14.77 to −0.23); *d* = 1.10	
**MI-PO**		−0.7 (−6.02 to 7.43); *d* = −0.12	2.72 (−3.52 to 8.95); *d* = −0.54	4.7 (−2.57 to 11.97); *d* = −0.79	
**AO-PO**		−4.1 (−2.63 to 10.82); *d* = −0.66	7.96 * (1.73 to 14.19); *d* = 1.4	12.2 ** (−19.47 to −4.93); *d* = 1.92	

* *p* < 0.05; ** *p* < 0.001. AO: action observation group; MI: motor imagery group; PO: placebo observation group; HR: heart rate; pre: preintervention measure; intervention: average intervention measure; post: postintervention measure.
